# Supplementary figures and images for: Aggression and discrimination among closely versus distantly related species of Drosophila
Source: R Soc Open Sci. 2019 Jun 12;6(6):190069. doi: 10.1098/rsos.190069 (PMC6599796; doi:10.1098/rsos.190069)

Supplemental Figure 1

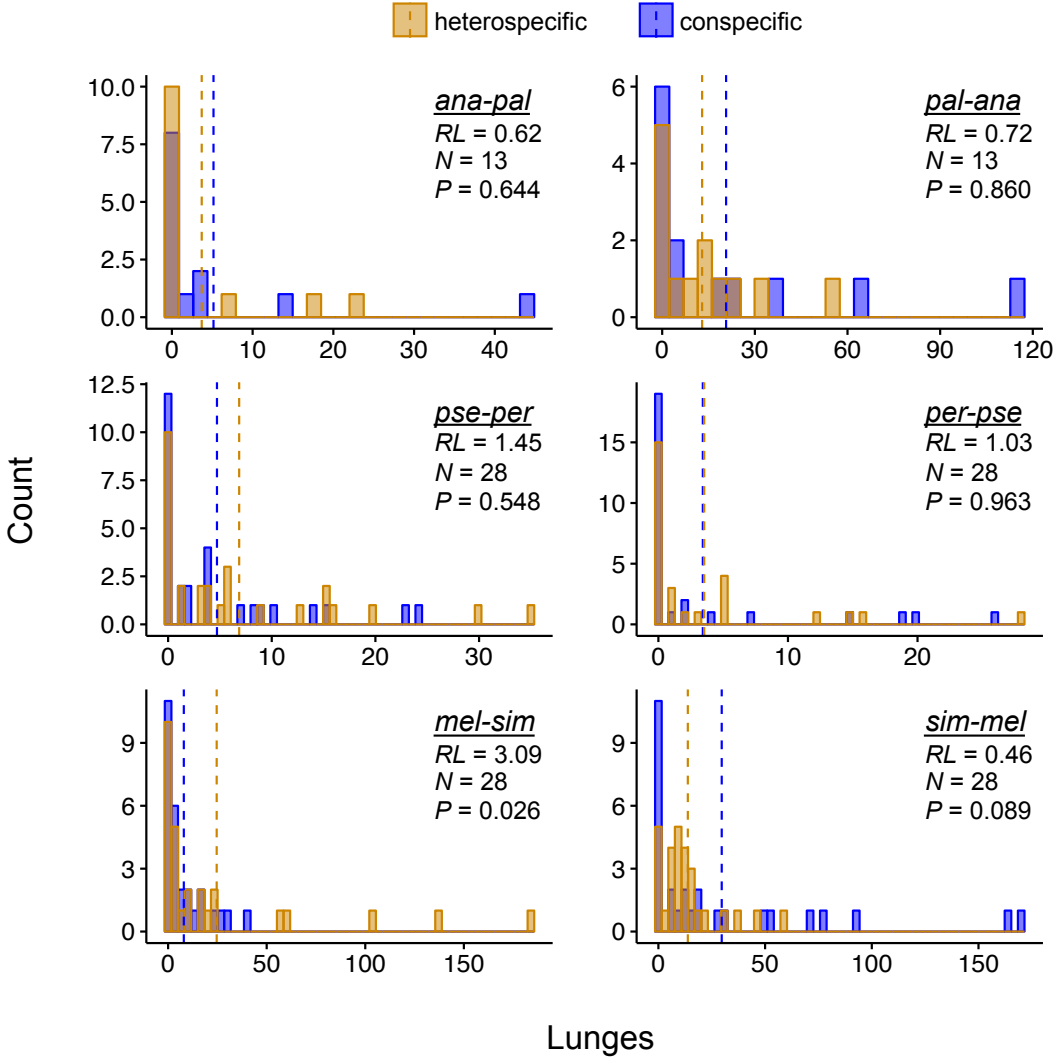

Supplement: Figure S1 [file rsos190069supp1.pdf]

Supplemental Figure 2

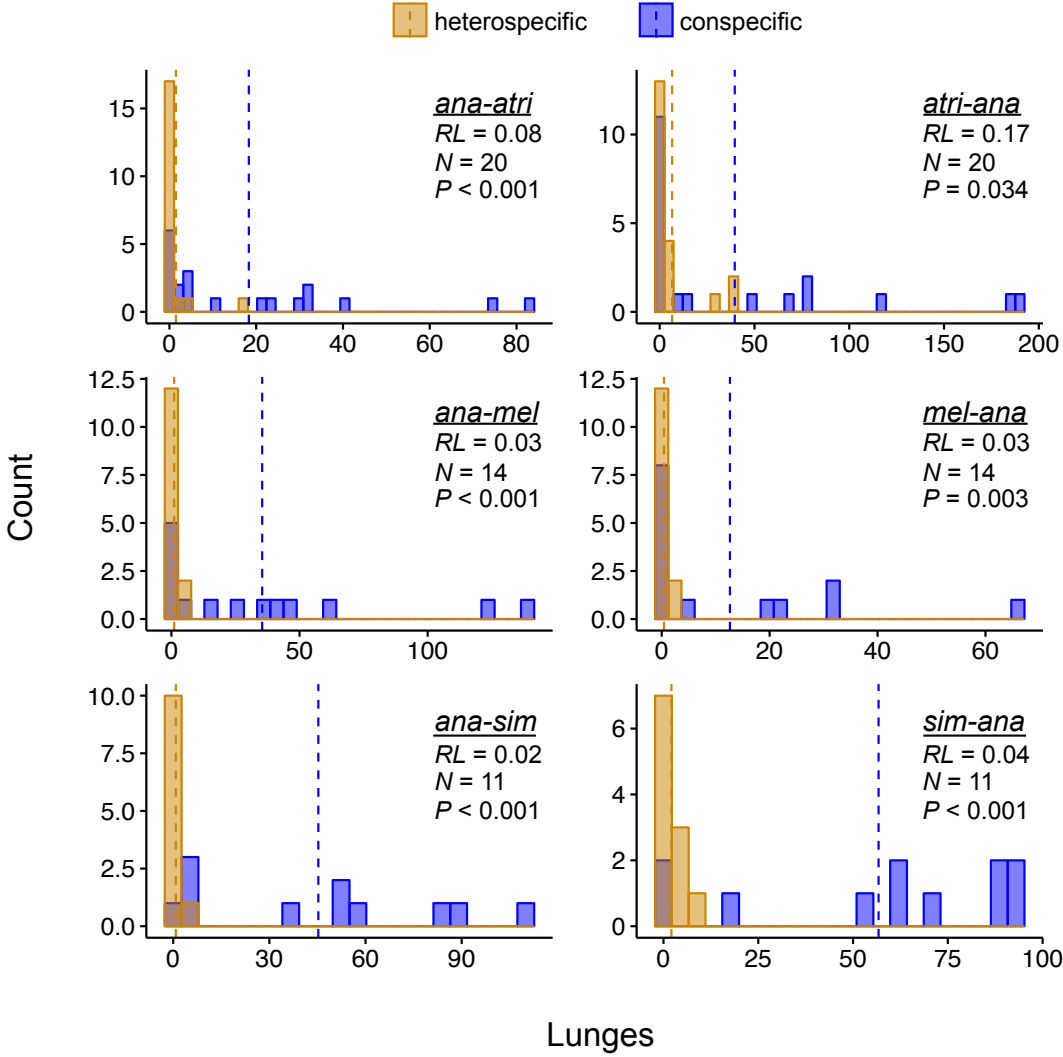

Supplement: Figure S2 [file rsos190069supp2.pdf]

**Supplemental Figure 3**

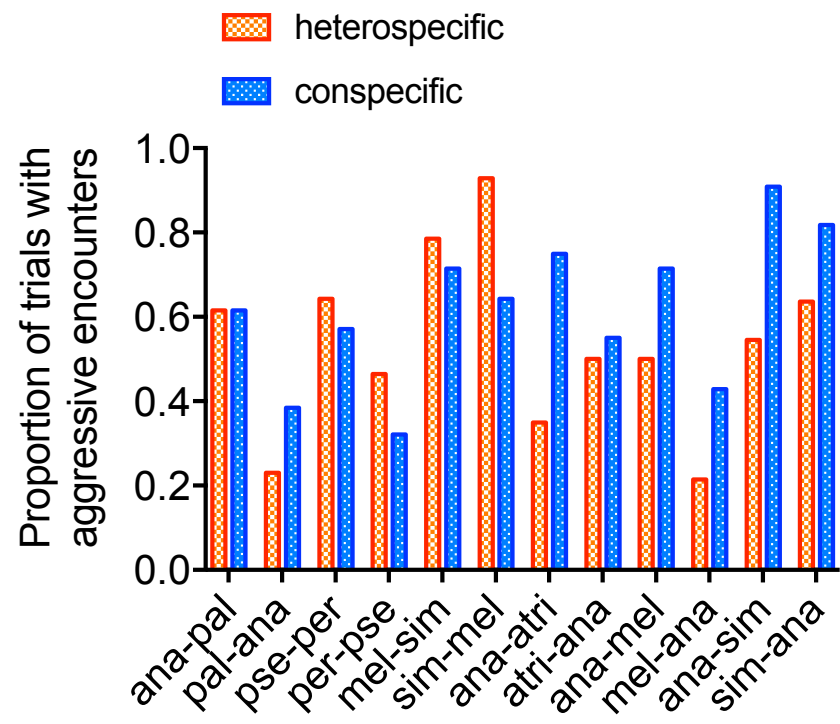

Supplement: Figure S3 [file rsos190069supp3.pdf]

Supplemental Figure 4

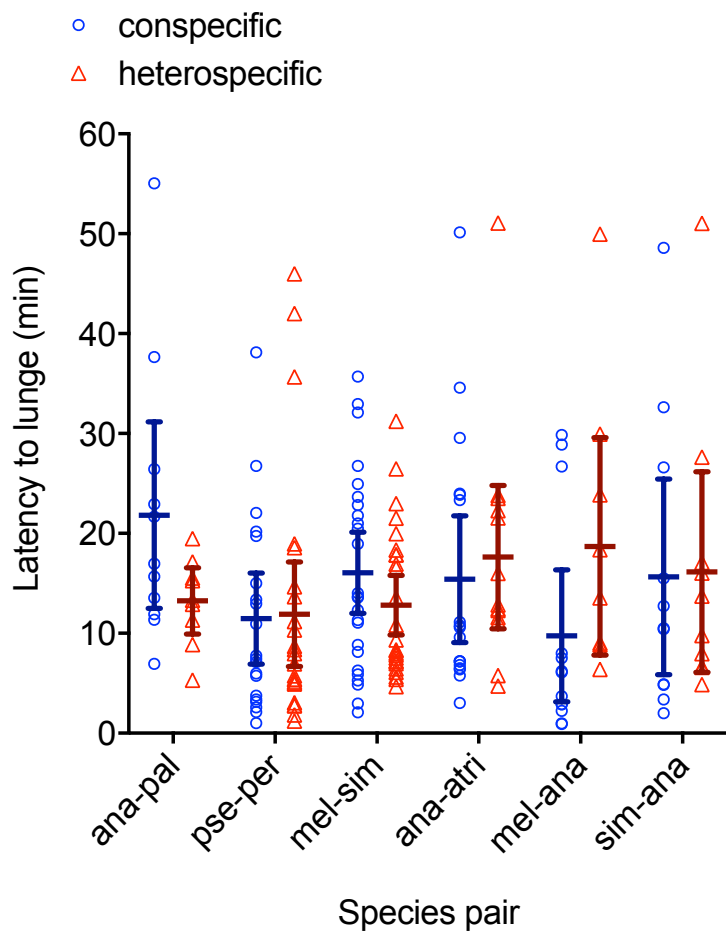

Supplement: Figure S4 [file rsos190069supp4.pdf]

# Supplemental Figure 5

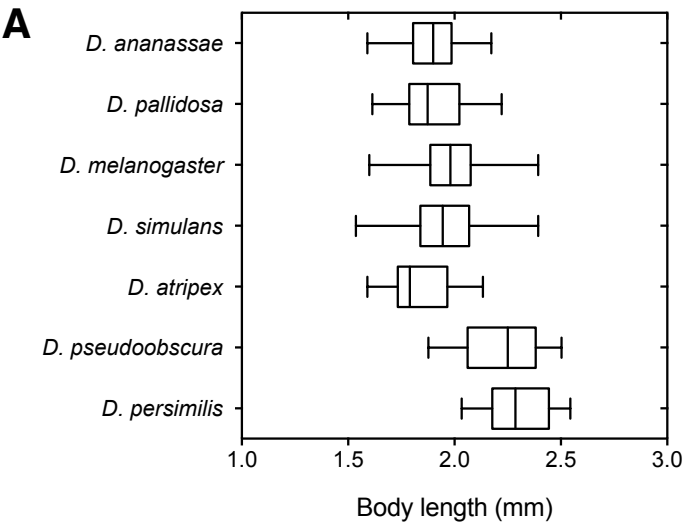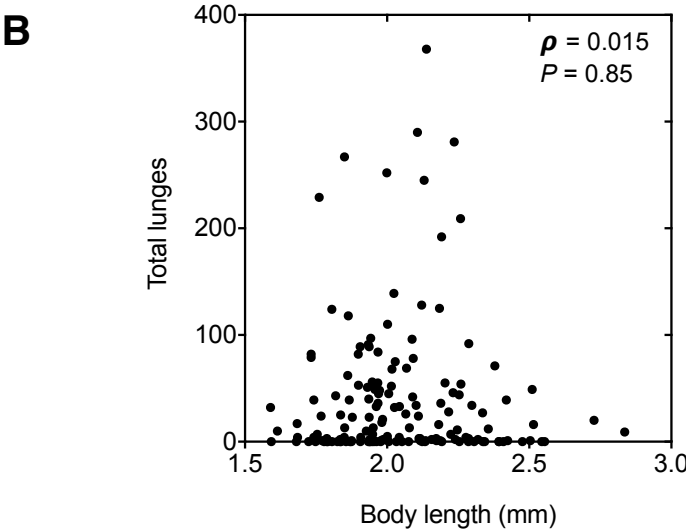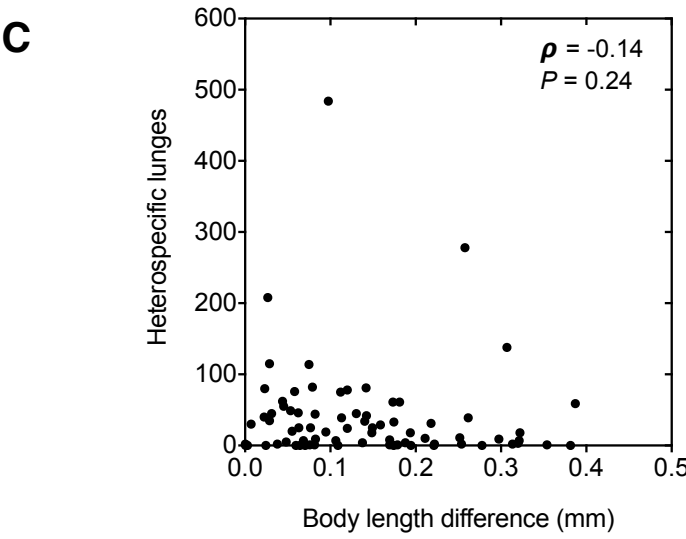

Supplement: Figure S5 [file rsos190069supp5.pdf]
